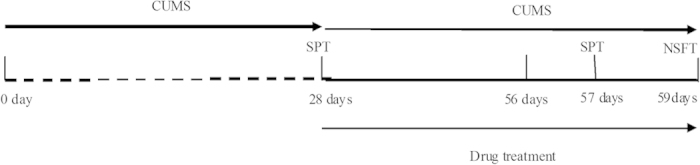# Fluoxetine regulates mTOR signalling in a region-dependent manner in depression-like mice

**DOI:** 10.1038/srep16024

**Published:** 2015-11-02

**Authors:** Xiao-Long Liu, Liu Luo, Rong-Hao Mu, Bin-Bin Liu, Di Geng, Qing Liu, Li-Tao Yi

**Affiliations:** 1 Department of Chemical and Pharmaceutical Engineering, College of Chemical Engineering, Huaqiao University, Xiamen 361021, Fujian province, PR China

## Abstract

Previous studies have demonstrated that the mammalian target of rapamycin (mTOR) signaling pathway has an important role in ketamine-induced, rapid antidepressant effects despite the acute administration of fluoxetine not affecting mTOR phosphorylation in the brain. However, the effects of long-term fluoxetine treatment on mTOR modulation have not been assessed to date. In the present study, we examined whether fluoxetine, a type of commonly used antidepressant agent, alters mTOR signaling following chronic administration in different brain regions, including the frontal cortex, hippocampus, amygdala and hypothalamus. We also investigated whether fluoxetine enhanced synaptic protein levels in these regions via the activation of the mTOR signaling pathway and its downstream regulators, p70S6K and 4E-BP-1. The results indicated that chronic fluoxetine treatment attenuated the chronic, unpredictable, mild stress (CUMS)-induced mTOR phosphorylation reduction in the hippocampus and amygdala of mice but not in the frontal cortex or the hypothalamus. Moreover, the CUMS-decreased PSD-95 and synapsin I levels were reversed by fluoxetine, and these effects were blocked by rapamycin only in the hippocampus. In conclusion, our findings suggest that chronic treatment with fluoxetine can induce synaptic protein expression by activating the mTOR signaling pathway in a region-dependent manner and mainly in the hippocampus.

Mammalian target of rapamycin (mTOR), a large serine/threonine kinase, regulates the initiation of protein translation in the body. mTOR acts as both a node of convergence downstream of the receptors, as well as a regulator of several signaling pathways^1^. Activation of mTOR results in its phosphorylation, thereby affecting its downstream effector molecules, activating p70S6 kinase and inhibiting 4E-binding proteins. These two molecules then control protein translation.

The mTOR signaling pathway integrates both intracellular and extracellular signals and controls the protein synthesis that is required for new synaptic connections^2^. It participates in hippocampus-dependent long-term memory consolidation^3^ and regulates new presynaptic or postsynaptic protein synthesis when required for neurogenesis^4^. Recent studies support the hypothesis that major depressive disorder might a consequence of a disruption in mTOR-dependent translation regulation. Therefore, deficits in the mTOR-dependent translation initiation pathway may contribute to the molecular and structural pathology of depression^5^. Thus, some studies hypothesize that depression results from deficits in synaptic proteins that are caused by abnormalities in mTOR signaling^6^. For this reason, the contribution of mTOR signaling to synaptic protein synthesis is currently a major research focus for depression.

Recent studies have demonstrated that rapid, antidepressant-like effects are associated with AMPA receptor-mediated up-regulation of mTOR in the rat frontal cortex and hippocampus^7^,^8^,^9^,^10^,^11^. However, research has also demonstrated that the acute administration of fluoxetine, a selective serotonin reuptake inhibitor antidepressant, improved the behaviors of and neurogenesis in rodents^12^, but did not reverse mTOR phosphorylation^7^. However, it is not known if mTOR phosphorylation is involved, via chronic fluoxetine administration, in the reversal of the depression-like behaviors, nor is it understood whether mTOR phosphorylation is necessary for synaptic protein expression. Therefore, in this study, we examined whether chronic fluoxetine treatment altered mTOR signaling in various brain regions, including the frontal cortex, hippocampus, amygdala and hypothalamus. Furthermore, because some studies have investigated the effect of antidepressant-induced mTOR signaling on synaptic protein levels^13^,^14^, we also investigated whether chronic fluoxetine treatment enhanced synaptic protein levels via the activation of the mTOR signaling pathway in these brain regions.

## Results

### Chronic fluoxetine treatment reverses chronic, unpredictable, mild stress (CUMS)-induced depressive symptoms

**Figure 1:**
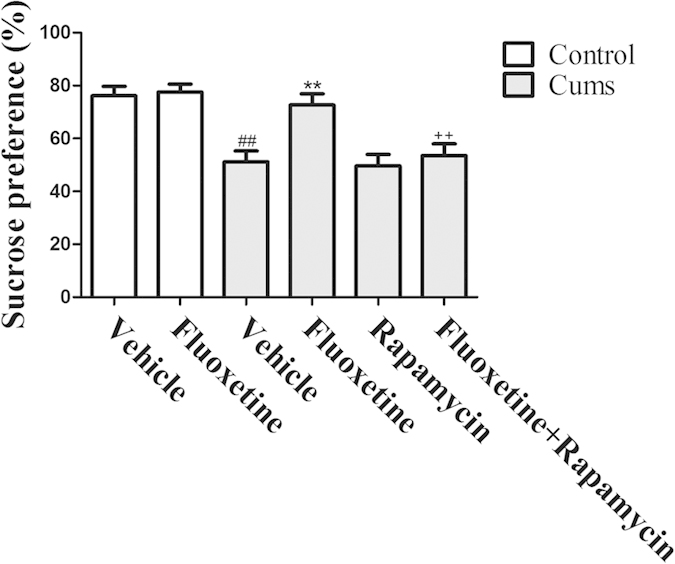
Effect of fluoxetine (20 mg/kg) and rapamycin (10 mg/kg) on sucrose preference in mice. The data represented the values of mean ± S.E.M. from 8 mice/group. ^##^*P* < 0.01 vs Control-vehicle group. **P < 0.01 vs CUMS-vehicle group. ^++^*P* < 0.01 vs CUMS-fluoxetine group. The results of Two-way ANOVA are provided in supplemental materials.

To evaluate whether mTOR signaling is necessary for the antidepressant-like effects of chronic fluoxetine treatment, mice that experienced CUMS were co-injected with fluoxetine (20 mg/kg) and rapamycin (10 mg/kg) for four weeks. Behavioral changes were examined 24 h after the final injection. As shown in Fig. 1, CUMS decreased sucrose preference [*F(*1, 14) = 20.86, *P* < 0.01] when compared with control group. Chronic treatment with fluoxetine for four weeks significantly reversed the CUMS-induced reduction in sucrose preference [*P* < 0.01]. In contrast, rapamycin blocked the antidepressant effect of fluoxetine [*P* < 0.01].

**Figure 2:**
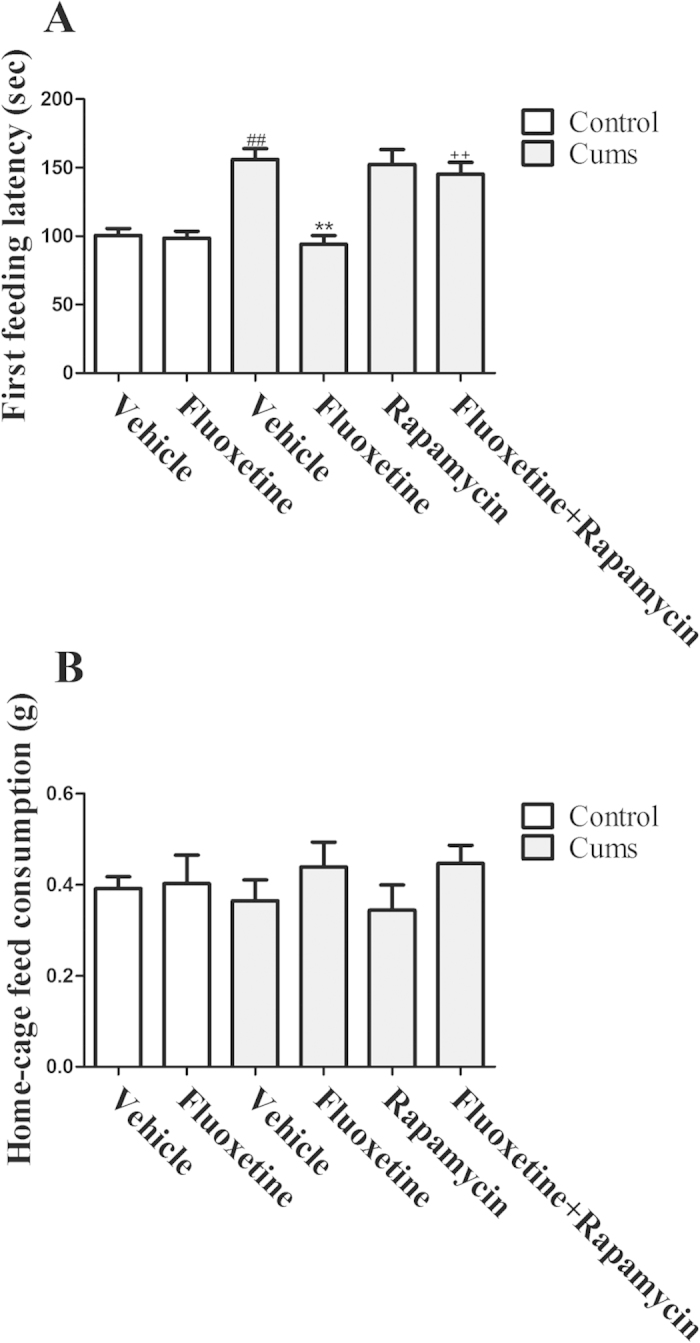
Effect of fluoxetine (20 mg/kg) and rapamycin (10 mg/kg) on the first feeding latency (**A**) and home-cage feeding consumption (**B**) in mice. The data represented the values of mean ± S.E.M. from 8 mice/group. ^##^*P* < 0.01 vs Control-vehicle group. **P < 0.01 vs CUMS-vehicle group. ^++^*P* < 0.01 vs CUMS-fluoxetine group. The results of Two-way ANOVA are provided in supplemental materials.

Additionally, CUMS increased the first feeding latency [*F(*1, 14) = 36.46, *P* < 0.01] in the novelty-suppressed feeding test, as shown in Fig. 2. Four weeks of chronic treatment with fluoxetine significantly reversed the CUMS-induced increase in the first feeding latency [*P* < 0.01], while rapamycin blocked the antidepressant effect of fluoxetine [*P* < 0.01]. However, there was no difference in the level of home-cage feed consumption immediately following the test within 5 min, which indicates that the effects of fluoxetine were not due to a general increase in feeding. These results demonstrated that rapamycin antagonized the antidepressant-like effects of fluoxetine.

### Chronic fluoxetine treatment attenuates the CUMS-induced mTOR phosphorylation reduction

**Figure 3:**
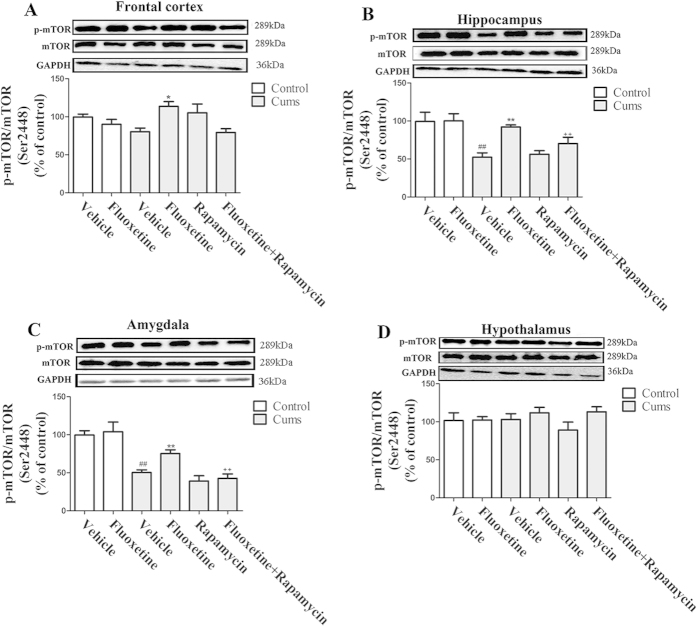
Effect of fluoxetine (20 mg/kg) and rapamycin (10 mg/kg) on the level of expression of phosphorylated-mTOR (Ser2448) protein in the frontal cortex (**A**), the hippocampus (**B**), the amygdala (**C**) and the hypothalamus (**D**). The data represented the values of the mean ± S.E.M. from 6 mice/group. ^##^*P* < 0.01 vs Control-vehicle group. ^*^P < 0.05 and **P < 0.01 vs CUMS-vehicle group. ^++^*P* < 0.01 vs CUMS-fluoxetine group. The results of Two-way ANOVA are provided in supplemental materials.

To further investigate the effects of fluoxetine on the modulation of mTOR phosphorylation in CUMS mice, we performed a western blot to determine the level of total and phosphorylated mTOR expression. As shown in Fig. 3, CUMS decreased mTOR phosphorylation in the hippocampus [*F(*1, 10) = 12.02, *P* < 0.01] (Fig. 3B) and the amygdala [*F(*1, 10) = 55.20, *P* < 0.01] (Fig. 3C). This effect was reversed by the administration of fluoxetine for four weeks [*P* < 0.01; *P* < 0.01, respectively] and that the effects of fluoxetine were blocked by rapamycin [*P* < 0.01; *P* < 0.01, respectively]. Furthermore, none of these effects were observed in the frontal cortex (Fig. 3A) or the hypothalamus (Fig. 3D). These results suggest that chronic fluoxetine treatment resulted in the activation of the mTOR signaling pathway in the hippocampus and amygdala, while rapamycin abolished its effects.

### Chronic fluoxetine treatment attenuates the CUMS-induced p70S6K phosphorylation reduction

**Figure 4:**
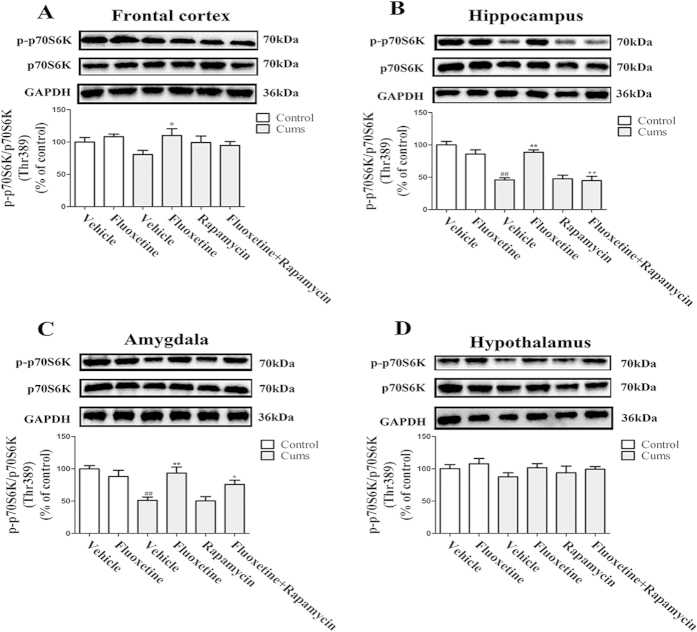
Effect of fluoxetine (20 mg/kg) and rapamycin (10 mg/kg) on the level of expression of phosphorylated-p70S6K expression in the frontal cortex (**A**), the hippocampus (**B**), the amygdala (**C**) and the hypothalamus (**D**). The data represented the values of mean ± S.E.M. from 6 mice/group. ^##^*P* < 0.01 vs Control-vehicle group. *P < 0.05 and **P < 0.01 vs CUMS-vehicle group. ^+^*P* < 0.05 and ^++^*P* < 0.01 vs CUMS-fluoxetine group. The results of Two-way ANOVA are provided in supplemental materials.

To investigate the effects of fluoxetine on the modulation of p70S6K phosphorylation in mice that were exposed to CUMS, we performed a western blot analysis to determine the levels of total and phosphorylated p70S6K expression. As shown in Fig. 4, CUMS decreased p70S6K phosphorylation in the hippocampus [*F(*1,10) = 92.67, *P* < 0.01] (Fig. 4B) and the amygdala [*F(*1, 10) = 67.69, *P* < 0.01] (Fig. 4C). In both instances, this decrease was reversed by the administration of fluoxetine for four weeks [*P* < 0.01; *P* < 0.01, respectively]. This itself was blocked by rapamycin [*P* < 0.01; *P* < 0.05, respectively]. Furthermore, none of these effects was seen in the frontal cortex (Fig. 4A) or the hypothalamus (Fig. 4D). These results suggest that chronic fluoxetine treatment activates p70S6K, which is a downstream regulator of mTOR in the hippocampus and amygdala, while rapamycin abolished its effects.

### Chronic fluoxetine treatment attenuates the CUMS-induced 4E-BP-1 phosphorylation reduction

**Figure 5:**
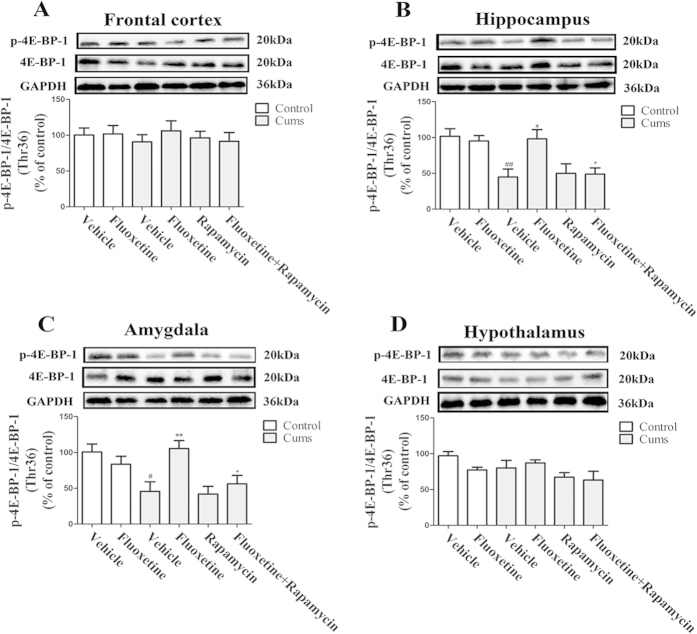
Effect of fluoxetine (20 mg/kg) and rapamycin (10 mg/kg) on the level of phosphorylated-4E-BP-1 expression in the frontal cortex (**A**), the hippocampus (**B**), the amygdala (**C**) and the hypothalamus (**D**). The data represented the values of mean ± S.E.M. from 5 mice/group. ^#^*P* < 0.05 and ^##^*P* < 0.01 vs Control-vehicle group. *P < 0.05 and **P < 0.01 vs CUMS-vehicle group. ^+^*P* < 0.05 vs CUMS-fluoxetine group. The results of Two-way ANOVA are provided in supplemental materials.

To investigate the effects of fluoxetine on the modulation of 4E-BP-1-protein phosphorylation in mice that were exposed to CUMS, we performed a western blot analysis to determine the levels of total and phosphorylated 4E-BP-1-protein expression. As shown in Fig. 5, CUMS decreased 4E-BP-1 phosphorylation in the hippocampus [*F(*1, 8) = 13.99, *P* < 0.01] (Fig. 5B) and the amygdala [*F(*1, 8) = 10.01, *P* < 0.05] (Fig. 5C). These effects were reversed by the administration of fluoxetine for four weeks [*P* < 0.05; *P* < 0.01, respectively], which itself was blocked by rapamycin [*P* < 0.05; *P* < 0.05, respectively]. Furthermore, none of these effects were observed in the frontal cortex (Fig. 5A) or the hypothalamus (Fig. 5D). These results suggest that chronic fluoxetine treatment activates 4E-BP-1, which is a downstream regulator of mTOR in the hippocampus and amygdals, while rapamycin abolished its effects.

### Chronic fluoxetine treatment attenuates the CUMS-induced PSD-95 reduction

**Figure 6:**
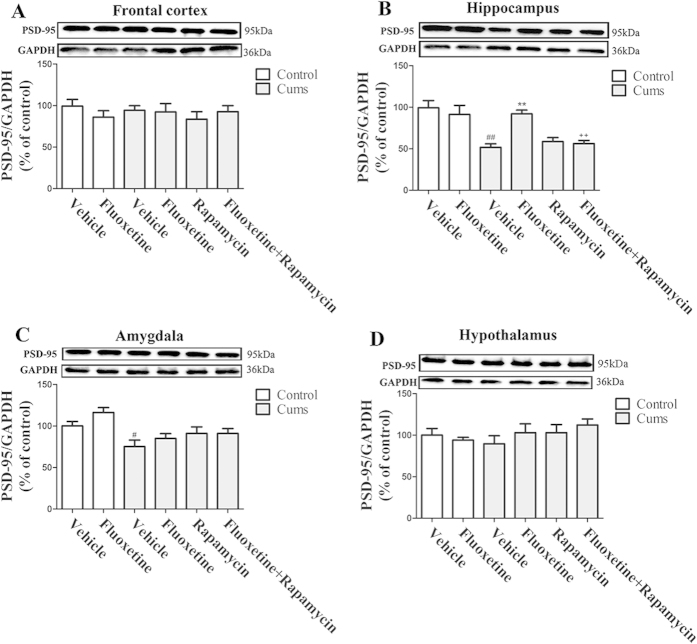
Effect of fluoxetine (20 mg/kg) and rapamycin (10 mg/kg) on the level of expression of PSD-95 protein in the frontal cortex (**A**), the hippocampus (**B**), the amygdala (**C**) and the hypothalamus (**D**). The data represented the values of the mean ± S.E.M. from 6 mice/group. ^#^*P* < 0.05 and ^##^*P* < 0.01 vs Control-vehicle group. **P < 0.01 vs CUMS-vehicle group. ^++^*P* < 0.01 vs CUMS-fluoxetine group. The results of Two-way ANOVA are provided in supplemental materials.

To examine the potential role of fluoxetine in the induction of postsynaptic protein expression in the hippocampus, we assessed PSD-95 expression in mice that were exposed to CUMS. As shown in Fig. 6, following eight weeks of exposure, CUMS decreased PSD-95 expression in the hippocampus [*F(*1, 10) = 23.98, *P* < 0.01] (Fig. 6B) and amygdala [*F(*1, 10) = 6.75, *P* < 0.05] (Fig. 6C). These effects in the hippocampus [*P* < 0.01] were reversed by the administration of fluoxetine for four weeks, which was then blocked by rapamycin in the hippocampus [*P* < 0.01]. Furthermore, none of these effects were seen in the frontal cortex (Fig. 6A) or the hypothalamus (Fig. 6D). These results suggest that chronic fluoxetine treatment up-regulated postsynaptic protein expression in the hippocampus, which was mediated by the mTOR signaling pathway.

### Chronic fluoxetine treatment attenuates the CUMS-induced synapsin I reduction

**Figure 7:**
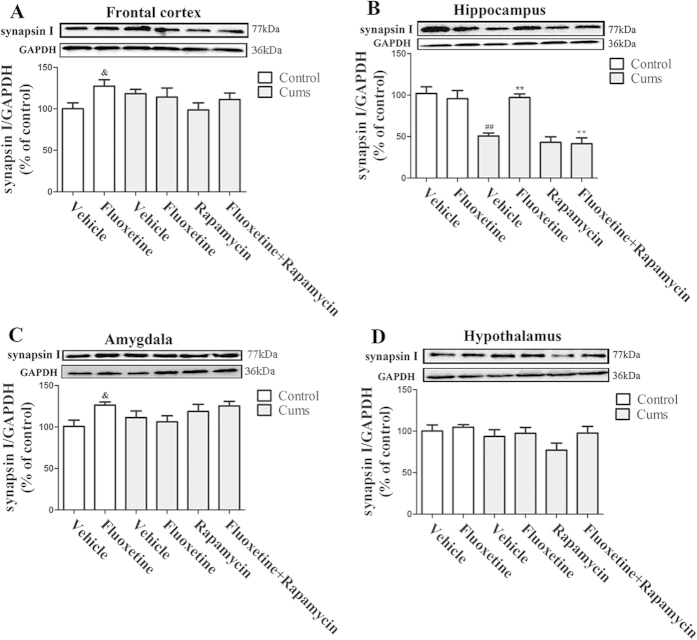
Effect of fluoxetine (20 mg/kg) and rapamycin (10 mg/kg) on the synapsin I protein expression in the frontal cortex (**A**), the hippocampus (**B**), the amygdala (**C**) and the hypothalamus (**D**). The data represented the values of the mean ± S.E.M. from 6 mice/group. ^##^*P* < 0.01 vs Control-vehicle group. ^**^P < 0.01 vs CUMS-vehicle group. ^++^*P* < 0.01 vs CUMS-fluoxetine group. ^&^*P* < 0.05 vs Control-vehicle group. The results of Two-way ANOVA are provided in supplemental materials.

To evaluate the potential role of fluoxetine in inducing presynaptic protein expression in the hippocampus, we assessed synapsin I expression in mice that were exposed to CUMS. As shown in Fig. 7, eight weeks of exposure to CUMS resulted in a decrease in synapsin I expression in the hippocampus [*F(*1, 10) = 33.65, *P* < 0.01] (Fig. 7B). Yet, this effect was reversed by the administration of fluoxetine for four weeks [*P* < 0.01], which itself was blocked by rapamycin treatment [*P* < 0.01]. Furthermore, none of these effects were observed in the frontal cortex (Fig. 7A), amygdala (Fig. 7C) or the hypothalamus (Fig. 7D). These results suggested that chronic fluoxetine treatment induced presynaptic protein expression in the hippocampus, which was mediated by the mTOR signaling pathway.

## Discussion

In the present study, we used an efficacious mouse CUMS model to mimic routinely encountered stress. The results indicated that a reduction in the sucrose preference that was induced by the CUMS procedure was significantly restored following chronic fluoxetine treatment. Subsequently, we performed the novelty-suppressed feeding test, an increasingly popular anxiety measure in the study of depression^15^. We found that chronic administration of fluoxetine reversed the prolonged first feeding latency that was induced by the CUMS. Conversely, the effects of fluoxetine were not due to a general increase in feeding. Thus, our present study suggests an antidepressant-like effect of fluoxetine.

The mTOR is a key signaling kinase that affects broad aspects of cellular functions, including metabolism, growth, survival, aging, synaptic plasticity and memory^16^. The effects of antidepressant agents in the rat forced swimming test were completely blocked by rapamycin, an mTOR inhibitor, which suggests that the antidepressant-like actions were mediated by activation of mTOR^17^. Some scientists have also demonstrated that some antidepressants significantly increase the levels of phosphorylated-mTOR as well as of its down-stream regulators (phosphorylated-4E-BP-1 and phosphorylated-p70S6K)^13^. The present study found that chronic fluoxetine treatment attenuated the CUMS-induced mTOR phosphorylation reduction in the hippocampus and the amygdala; however, in the frontal cortex and the hypothalamus, neither CUMS nor fluoxetine altered the level of mTOR phosphorylation. A recent study also demonstrated that CUMS affects mTOR phosphorylation in different brain regions^18^. Yet, this paper reported that the CUMS-induced decrease in mTOR phosphorylation only occurred in the amygdala and not in the hippocampus. In fact, CUMS-induced mTOR phosphorylation is equivocal, because some studies have reported that the CUMS significantly decreased mTOR phosphorylation in the hippocampus^10^,^11^. Thus, variations in the level of mTOR phosphorylation that is induced by CUMS may depend on the experimental stressors, animals, behavioral tests, hippocampal homogenates and, the time point following vehicle/agent administration that are adopted by different experimenters^19^.

Meanwhile, previous studies have shown that the inhibition of mTOR by rapamycin reversed the antidepressant-like effects of ketamine in the depression-like animals^8^,^20^. To confirm that the antidepressant-like effects of chronic fluoxetine treatment were mediated by mTOR signaling, we injected rapamycin prior to fluoxetine administration. The results demonstrated that rapamycin not only blocked the behavioral improvement that was induced by fluoxetine but also abolished the mTOR phosphorylation following CUMS. These results indicate a possible involvement of the antidepressant-like effects of fluoxetine in mTOR signaling changes.

mTOR has an important role in cell growth and proliferation by phosphorylating p70 ribosomal protein S6 kinase (p70S6K) and eukaryotic initiation factor 4E-binding protein 1 (4E-BP-1), which subsequently promotes ribosomal biogenesis and the mRNA translation of proteins that are required for cell proliferation^21^. The present study found that chronic fluoxetine treatment reversed the CUMS-induced reduction of phosphorylation of p70S6K and 4E-BP-1 in the hippocampus and the amygdala. Yet, in the frontal cortex and the hypothalamus, neither CUMS nor fluoxetine altered the level of phosphorylation of p70S6K and 4E-BP-1. The variations in the levels of phosphorylation of p70S6K and 4E-BP-1 were consistent with the changes of mTOR phosphorylation. A previous study has shown that the phosphorylated and activated forms of mTOR, p70S6K and 4E-BP-1 were observed in a preparation that was enriched with synaptoneurosomes^7^. The immobilization stress significantly decreased the phosphorylation of mTOR and p70S6K in the hippocampus of rats^13^. Meanwhile, many antidepressants significantly increased the levels of phosphorylated-mTOR and of its downstream regulators (phospho-p70S6K and phospho-4E-BP-1)^22^. Based on these studies, one could hypothesize that the activation of mTOR signaling increases the phosphorylation of p70S6K and 4E-BP-1, thereby promoting the initiation of protein translation for synaptic protein synthesis.

Because PSD-95 and synapsin I are important for synapse formation, we measured the changes of these two proteins. They participate in the formation and maintenance of synaptic contacts among the central neurons^23^. As a result, synapsins are used as markers of presynaptic terminals. In the present study, chronic fluoxetine treatment attenuated the CUMS-induced reduction in PSD-95 and synapsin I levels, while rapamycin abolished the effect of fluoxetine in the hippocampus. Recent studies have demonstrated that the rapid antidepressant response to ketamine was mediated by the activation of the mTOR-signaling pathway, thereby leading to an increased level of synaptic proteins in rats^8^. Moreover, several core components of the mTOR signaling pathway were present in dendrites^24^, and the involvement of mTOR signaling in synaptic protein synthesis has been recently characterized^25^. These studies might indicate an association between the synthesis of PSD-95, synapsin I and mTOR signaling. Considering that chronic fluoxetine treatment attenuated the CUMS-induced reduction in mTOR phosphorylation, the changes of PSD-95 and synapsin I protein levels are evidence of an association between the antidepressant-like effects of fluoxetine and mTOR regulation. Therefore, we speculate that the synthesis of synaptic proteins is mediated by the mTOR signaling pathway and its downstream regulators, p70S6K and 4E-BP-1, in the hippocampus.

The present results indicated that, in the hippocampus, CUMS decreased PSD-95 and synapsin I levels, while chronic fluoxetine treatment attenuated the reduction, which itself was abolished by treatment with rapamycin. However, this was not observed in the frontal cortex, the amygdala or the hypothalamus. These observations are paralleled in other studies. For example, chronic fluoxetine treatment reverses hippocampal PSD-95 reduction that is induced by learned helplessness^26^. The up-regulated effects of the mTOR signaling pathway are associated with an increase in hippocamal synapsins^27^. In contrast to the findings in the hippocampus of CUMS-treated mice, fluoxetine treatment did not alter the expression of any of the synaptic proteins in the frontal cortex^28^. Furthermore, the expression of synapse-associated proteins, such as PSD-95 and synapsin, were significantly decreased in the lateral amygdala of CUMS-treated rats^29^. While the reason for discrepancies between these studies on the effect of chronic fluoxetine treatment on total PSD-95 and synapsin I protein levels in different brain regions is not clear, it may be a result of different treatment schedules, which ranges from 7 to 28 days, as well as of variation in the types of stressors, animal species, or gender used.

In contrast to our finding that chronic fluoxetine treatment attenuated the CUMS-induced PSD-95 and synapsin I reduction and that rapamycin abolished the effect of fluoxetine in the hippocampus of mice, a recent study demonstrated that rapamycin did not block the effect of fluoxetine on the synthesis of PSD-95 and synapsin I in rat hippocampal neurons^13^. The simplest interpretation of this finding is that the effect of antidepressant drugs varies between cell culture and brain tissue. The concentrations of antidepressant drugs that were used to produce positive effects are generally higher than the concentrations normally observed in the plasma. This discrepancy might be attributable to the different conditions between cell culture and the brain tissue.

In summary, the results of our present study suggeste that chronic fluoxetine treatment attenuates the CUMS-induced reduction in phosphorylation of mTOR and of its downstream regulators p70S6K and 4E-BP1 in the hippocampus and the amygdala, but not in the frontal cortex or the hypothalamus, of mice. Chronic fluoxetine treatment only attenuated the CUMS-induced reduction in expression of PSD-95 and synapsin I in the hippocampus. Furthermore, rapamycin abolished the effect of fluoxetine. Although these findings cannot conclusively demonstrate a relationship between fluoxetine-induced changes in synaptic protein expression and the mTOR signaling pathway in the regions that we tested, we hypothesize that chronic treatment with fluoxetine can induce synaptic protein expression by activating the mTOR signaling pathway and its downstream regulators, p70S6K and 4E-BP1, in the hippocampus. Additional studies are required to investigate other specific components of the signaling pathway that are involved in the synaptic protein-induced effect of fluoxetine.

## Methods

### Animals

Male ICR mice (24 ± 2 g; 5 weeks old) were purchased from the Laboratory Animal Centre, Fujian Medical University, Fujian Province, PR China. Eight animals were housed per cage (320 × 180 × 160 mm) with a normal 12-h/12-h light/dark schedule and with the lights on at 07:00 a.m. The animals were allowed one week to acclimate to the housing conditions prior to the beginning of experiments. The ambient temperature and relative humidity were maintained at 22 ± 2 °C and 55 ± 5%, respectively, and the animals were given standard chow and water *ad libitum* for the duration of the study. All procedures were approved by the Institute for Experimental Animals and were performed in accordance with the published guidelines of the China Council on Animal Care (Regulations for the Administration of Affairs Concerning Experimental Animals, approved by the State Council on 31 October, 1988 and promulgated by Decree No. 2 of the State Science and Technology Commission on 14 November, 1988).

### Drugs and reagents

The selective serotonin reuptake inhibitor fluoxetine was purchased from Sigma-Aldrich (St. Louis, USA). Rapamycin was purchased from MedChemexpress CO., Ltd (Monmouth Junction, USA). The antibodies for mTOR, phosphorylated-mTOR, p70S6K, phosphorylated- p70S6K, PSD-95 and synapsin I were purchased from Cell Signaling Technology (Beverly, USA). The antibodies for 4E-BP-1 and phosphorylated-4E-BP-1 were purchased form Affinity Biosciences (Cincinnati, USA). The anti-GAPDH antibody was purchased from Kangcheng Biotech (Shanghai, PR China).

### Drug administration

To investigate whether the mTOR signaling pathway is required for the antidepressant-like effect of chronic fluoxetine treatment, mice were randomly divided into six groups: a control-vehicle group, a control-fluoxetine group (20 mg/kg, p.o.), a CUMS-vehicle group, a CUMS-fluoxetine group (20 mg/kg, p.o.), a CUMS-rapamycin group (10 mg/kg, i.p.), and a CUMS-fluoxetine + rapamycin group (20 mg/kg, p.o. + 10 mg/kg, i.p.). All agents were dissolved in 1% DMSO and were administered in a volume of 10 mL/kg. After four weeks of CUMS exposure, the animals were administered with fluoxetine once a day over a four-week period. For pretreatment, animals first received either a vehicle or a rapamycin treatment once every third day, which was followed 30 minutes later by the administration of fluoxetine (Fig. 8).

The treatment doses and routs were selected on the basis of the behavioral results and previous reports^30^,^31^. The repeated drug treatment was performed once daily for the last four weeks of the experiment.

### CUMS

The CUMS procedure was performed as previously described^31^. Briefly, the weekly stress regime consisted of food and water deprivation, exposure to an empty bottle, exposure to a soiled cage, light/dark succession every 2 h, space reduction, 45° cage tilt, overnight illumination, and predator sounds. All stressors were applied individually and continuously during both day and night. The control animals were housed in a separate room and had no contact with the stressed groups. To prevent habituation and to ensure the unpredictability of the stressors, all stressors were randomly scheduled over a one-week period and were repeated throughout the eight-week experiment. On the basis of their sucrose preference following four weeks of CUMS, both stressed and control mice were divided into matched subgroups.

### Sucrose preference test

The sucrose preference test was conducted at the end of the fourth and eighth week of CUMS exposure. Briefly, prior to the test, the mice were trained to adapt to the sucrose solution (1%, w/v): two bottles of sucrose solution were placed in each cage for 24 h, and then one bottle of sucrose solution was replaced with water for 24 h. After the adaptation, the mice were deprived of water and food for 24 h. The test was conducted at 9:30 a.m. The mice were housed in individual cages and had free access to two bottles, one containing sucrose solution and the other containing water. After 24 h, the volumes of the consumed sucrose solution and water were recorded.

### Novelty-suppressed feeding test

The novelty-suppressed feeding test was performed 48 h after the last sucrose preference test and during a 8-min period, as previously described^32^,^33^. Briefly, the testing apparatus consisted of a plastic box (50 × 50 × 20 cm). Food was withheld from the mice for 24 h prior to the test. At the beginning of the test, a single pellet of food was placed on a white paper platform positioned at the center of the box. A mouse was placed in a corner of the maze box and a stopwatch was immediately started. Scoring to measure interest did not begin until the mouse reached for the food with its forepaws and began eating. The home-cage food consumption within 5 min was measured immediately following the test as a control value.

### Tissue sample collection

Ten hours after the completion of the novelty-suppressed feeding test, the mice were sacrificed by decapitation. Whole brains were rapidly removed from the mice and chilled in an ice-cold saline solution. The frontal cortex, the hippocampus, the amygdala and the hypothalamus were dissected on a cold plate and immediately frozen in liquid nitrogen. The tissue samples were stored at −80 °C until measured by an assay.

### Western blot

Brain samples were homogenized in a lysis buffer and were incubated on ice for 30 min. The homogenates were centrifuged at 14000 × *g* for 20 min at 4 °C. The supernatants were then collected. The protein concentration was determined by a BCA assay. The proteins were separated using a SDS-PAGE gel and were then transferred to a PVDF membrane. Following blocking in a 3% BSA/TBST solution at room temperature for 1 h, the membranes were incubated with the appropriate primary antibodies at 4 °C overnight (anti-PSD-95: 1:1000, anti-synapsin-1: 1:1000, anti-mTOR: 1:1000, anti-phosphorylated-mTOR: 1:1000, anti-p70S6k: 1:1000, anti-phosphorylated-p70S6k: 1:1000, anti-4E-BP-1: 1:1000, anti-phosphorylated-4E-BP-1: 1:1000, or anti-GAPDH: 1:5000). After the membranes were washed three times with TBST, the membranes were incubated with an HRP-labeled secondary antibody (1:1000). The blots were washed again three times with TBST buffer, and the immunoreactive bands were detected using an enhanced chemiluminescence method. The results were normalized to the protein expression level of GAPDH in each sample.

### Statistical analyses

All data were analyzed using SPSS software 13.0 (SPSS Inc., Chicago, USA) and expressed as the mean ± S.E.M. The data were analyzed using two two-way ANOVAs. The first included the Control/CUMS and Vehicle/Fluoxetine groups while the second included the Vehicle/Fluoxetine and Rapamycin/Rapamycin + Fluoxetine groups from the four CUMS groups. These were then followed by the Tukey’s *post-hoc* test to verify the hypotheses that CUMS will induce various effects that can be reversed by fluoxetine, a treatment that itself is reversed by rapamycin. A *P*-value < 0.05 was considered to be statistically significant for analysis.

### Role of the funding source

Funding for this study was provided by the National Natural Science Foundation of China (No. 81202940), the Science Research Foundation of ministry of Health & United Fujian Provincial Health and Education Project for Tacking the Key Research (WKJ-FJ-31) and the Promotion Program for Young and Middle-aged Teacher in Science and Technology Research of Huaqiao University (ZQN-PY218); the funders had no further role in study design; in the collection, analysis and interpretation of data; in the writing of the report; and in the decision to submit the paper for publication.

## Additional Information

**How to cite this article**: Liu, X.-L. *et al.* Fluoxetine regulates mTOR signalling in a region-dependent manner in depression-like mice. *Sci. Rep.* **5**, 16024; doi: 10.1038/srep16024 (2015).

## Supplementary Material

Supplementary Information

## Figures and Tables

**Figure 8:**