# Supplementary material for: Fluoxetine regulates mTOR signalling in a region-dependent manner in depression-like mice
Source: Sci Rep. 2015 Nov 2;5:16024. doi: 10.1038/srep16024 (PMC4629199; doi:10.1038/srep16024)
Supplement: Supplementary Information [file srep16024-s1.doc]

**Fluoxetine regulates mTOR signalling in a region-dependent manner in depression-like mice**

Xiao-Long Liu, Liu Luo, Rong-Hao Mu, Bin-Bin Liu, Di Geng, Qing Liu, Li-Tao Yi**^*^**

**Affiliation**

*Department of Chemical and Pharmaceutical Engineering, College of Chemical Engineering, Huaqiao University, Xiamen 361021, Fujian province, PR China*

*Corresponding author. Tel.: 86-592-6162302; Fax: 86-592-6162302.

E-mail address: litao.yi@yahoo.com; litaoyi@hqu.edu.cn (Li-Tao Yi)

**Short title: Fluoxetine regulates mTOR signalling**

**Fig.1.** The two-way ANOVA (stress and treatment) results of sucrose preference: stress [*F(*1,28)=15.54, *P*<0.01], treatment [*F(*1,28)=9.11, *P*<0.01], interaction [*F(*1,28)=7.05, *P*<0.05]. Another two-way ANOVA (treatment and pretreatment) results of sucrose preference: treatment [*F(*1,28)=8.65, *P*<0.01], pretreatment [*F(*1,28)=5.72, *P*<0.05], interaction [*F(*1,28)=4.18, *P*>0.05].

**Fig.2.** The two-way ANOVA (stress and treatment) results of the first feeding latency: stress [*F(*1,28)=17.66, *P*<0.01], treatment [*F(*1,28)=27.81, *P*<0.01], interaction [*F(*1,28)=24.44, *P*<0.01]. Another two-way ANOVA (treatment and pretreatment) results of the first feeding latency: treatment [*F(*1,28)=16.36, *P*<0.01], pretreatment [*F(*1,28)=7.68, *P*<0.05], interaction [*F(*1,28)=10.31, *P*<0.01].

**Fig.3.** (A) The two-way ANOVA (stress and treatment) results of mTOR phosphorylation in frontal cortex: stress [*F(*1,20)=0.16, *P*>0.05], treatment [*F(*1,20)=4.61, *P*<0.05], interaction [*F(*1,20)=15.23, *P*<0.01]. Another two-way ANOVA (treatment and pretreatment) results of mTOR phosphorylation in frontal cortex: treatment [*F(*1,20)=0.57, *P*>0.05], pretreatment [*F(*1,20)=0.92, *P*>0.05], interaction [*F(*1,20)=37.30, *P*<0.01]. (B) The two-way ANOVA (stress and treatment) results of mTOR phosphorylation in hippocampus: stress [*F(*1,20)=9.04, *P*<0.01], treatment [*F(*1,20)=6.46, *P*<0.05], interaction [*F(*1,20)=6.07, *P*<0.05]. Another two-way ANOVA (treatment and pretreatment) results of mTOR phosphorylation in hippocampus: treatment [*F(*1,20)=21.70, *P*<0.01], pretreatment [*F(*1,20)=4.46, *P*<0.05], interaction [*F(*1,20)=7.88, *P*<0.05]. (C) The two-way ANOVA (stress and treatment) results of mTOR phosphorylation in amygdala: stress [*F(*1,20)=22.98, *P*<0.01], treatment [*F(*1,20)=5.08, *P*<0.05], interaction [*F(*1,20)=2.84, *P*>0.05]. Another two-way ANOVA (treatment and pretreatment) results of mTOR phosphorylation in amygdala: treatment [*F(*1,20)=13.68, *P*<0.01], pretreatment [*F(*1,20)=29.00, *P*<0.01], interaction [*F(*1,20)=8.38, *P*<0.01]. (D) The two-way ANOVA (stress and treatment) results of mTOR phosphorylation in hypothalamus: stress [*F(*1,20)=1.23, *P*>0.05], treatment [*F(*1,20)=0.94, *P*>0.05], interaction [*F(*1,20)=0.69, *P*>0.05]. Another two-way ANOVA (treatment and pretreatment) results of mTOR phosphorylation in hypothalamus: treatment [*F(*1,20)=3.62, *P*>0.05], pretreatment [*F(*1,20)=2.48, *P*>0.05], interaction [*F(*1,20)=0.03, *P*>0.05].

**Fig.4.** (A) The two-way ANOVA (stress and treatment) results of p70S6K phosphorylation in frontal cortex: stress [*F(*1,20)=1.73, *P*>0.05], treatment [*F(*1,20)=8.09, *P*<0.05], interaction [*F(*1,20)=2.53, *P*>0.05]. Another two-way ANOVA (treatment and pretreatment) results of p70S6K phosphorylation in frontal cortex: treatment [*F(*1,20)=4.15, *P*>0.05], pretreatment [*F(*1,20)=0.02, *P*>0.05], interaction [*F(*1,20)=6.09, *P*<0.05]. (B) The two-way ANOVA (stress and treatment) results of p70S6K phosphorylation in hippocampus: stress [*F(*1,20)=61.70, *P*<0.01], treatment [*F(*1,20)=18.55, *P*<0.01], interaction [*F(*1,20)=75.09, *P*<0.01]. Another two-way ANOVA (treatment and pretreatment) results of p70S6K phosphorylation in hippocampus: treatment [*F(*1,20)=24.66, *P*<0.01], pretreatment [*F(*1,20)=27.19, *P*<0.01], interaction [*F(*1,20)=31.67, *P*<0.01]. (C) The two-way ANOVA (stress and treatment) results of p70S6K phosphorylation in amygdala: stress [*F(*1,20)=34.20, *P*<0.01], treatment [*F(*1,20)=16.81, *P*<0.01], interaction [*F(*1,20)=52.38, *P*<0.01]. Another two-way ANOVA (treatment and pretreatment) results of p70S6K phosphorylation in amygdala: treatment [*F(*1,20)=54.45, *P*<0.01], pretreatment [*F(*1,20)=4.53, *P*<0.05], interaction [*F(*1,20)=2.55, *P*>0.05]. (D) The two-way ANOVA (stress and treatment) results of p70S6K phosphorylation in hypothalamus: stress [*F(*1,20)=2.73, *P*>0.05], treatment [*F(*1,20)=3.80, *P*>0.05], interaction [*F(*1,20)=0.37, *P*>0.05]. Another two-way ANOVA (treatment and pretreatment) results of p70S6K phosphorylation in hypothalamus: treatment [*F(*1,20)=5.91, *P*<0.05], pretreatment [*F(*1,20)=0.22, *P*>0.05], interaction [*F(*1,20)=1.17, *P*>0.05].

**Fig.5.** (A) The two-way ANOVA (stress and treatment) results of 4E-BP-1 phosphorylation in frontal cortex: stress [*F(*1,16)=0.05, *P*>0.05], treatment [*F(*1,16)=0.55, *P*>0.05], interaction [*F(*1,16)=0.35, *P*>0.05]. Another two-way ANOVA (treatment and pretreatment) results of 4E-BP-1 phosphorylation in frontal cortex: treatment [*F(*1,16)=0.21, *P*>0.05], pretreatment [*F(*1,16)=0.15, *P*>0.05], interaction [*F(*1,16)=0.76, *P*>0.05]. (B) The two-way ANOVA (stress and treatment) results of 4E-BP-1 phosphorylation in hippocampus: stress [*F(*1,16)=6.31, *P*<0.05], treatment [*F(*1,16)=4.66, *P*<0.05], interaction [*F(*1,16)=7.79, *P*<0.05]. Another two-way ANOVA (treatment and pretreatment) results of 4E-BP-1 phosphorylation in hippocampus: treatment [*F(*1,16)=4.93, *P*<0.05], pretreatment [*F(*1,16)=3.53, *P*>0.05], interaction [*F(*1,16)=5.31, *P*<0.05]. (C) The two-way ANOVA (stress and treatment) results of 4E-BP-1 phosphorylation in amygdala: stress [*F(*1,16)=2.02, *P*>0.05], treatment [*F(*1,16)=3.35, *P*>0.05], interaction [*F(*1,16)=10.79, *P*<0.01]. Another two-way ANOVA (treatment and pretreatment) results of 4E-BP-1 phosphorylation in amygdala: treatment [*F(*1,16)=9.94, *P*<0.01], pretreatment [*F(*1,16)=5.07, *P*<0.05], interaction [*F(*1,16)=3.72, *P*>0.05]. (D) The two-way ANOVA (stress and treatment) results of 4E-BP-1 phosphorylation in hypothalamus: stress [*F(*1,16)=0.08, *P*>0.05], treatment [*F(*1,16)=0.16, *P*>0.05], interaction [*F(*1,16)=1.07, *P*>0.05]. Another two-way ANOVA (treatment and pretreatment) results of 4E-BP-1 phosphorylation in hypothalamus: treatment [*F(*1,16)=0.14, *P*>0.05], pretreatment [*F(*1,16)=1.97, *P*>0.05], interaction [*F(*1,16)=0.07, *P*>0.05].

**Fig.6.** (A) The two-way ANOVA (stress and treatment) results of PSD-95 expression in frontal cortex: stress [*F(*1,20)=0.01, *P*>0.05], treatment [*F(*1,20)=2.27, *P*>0.05], interaction [*F(*1,20)=1.24, *P*>0.05]. Another two-way ANOVA (treatment and pretreatment) results of PSD-95 expression in frontal cortex: treatment [*F(*1,20)=3.21, *P*>0.05], pretreatment [*F(*1,20)=0.03, *P*>0.05], interaction [*F(*1,20)=4.71, *P*<0.05]. (B) The two-way ANOVA (stress and treatment) results of PSD-95 expression in hippocampus: stress [*F(*1,20)=9.38, *P*<0.01], treatment [*F(*1,20)=4.44, *P*<0.05], interaction [*F(*1,20)=9.92, *P*<0.05]. Another two-way ANOVA (treatment and pretreatment) results of PSD-95 expression in hippocampus: treatment [*F(*1,20)=19.13, *P*<0.01], pretreatment [*F(*1,20)=10.89, *P*<0.01], interaction [*F(*1,20)=24.12, *P*<0.01]. (C) The two-way ANOVA (stress and treatment) results of PSD-95 expression in amygdala: stress [*F*(1,20)=19.91, *P*<0.01], treatment [*F*(1,20)=4.29, *P*>0.05], interaction [*F*(1,20)=0.28, *P*>0.05]. Another two-way ANOVA (treatment and pretreatment) results of PSD-95 expression in amygdala: treatment [*F*(1,20)=0.49, *P*>0.05], pretreatment [*F*(1,20)=2.44, *P*>0.05], interaction [*F*(1,20)=0.49, *P*>0.05]. (D) The two-way ANOVA (stress and treatment) results of PSD-95 expression in hypothalamus: stress [*F*(1,20)=0.02, *P*>0.05], treatment [*F*(1,20)=0.47, *P*>0.05], interaction [*F*(1,20)=3.27, *P*>0.05]. Another two-way ANOVA (treatment and pretreatment) results of PSD-95 expression in hypothalamus: treatment [*F*(1,20)=3.60, *P*>0.05], pretreatment [*F*(1,20)=3.59, *P*>0.05], interaction [*F*(1,20)=0.11, *P*>0.05].

**Fig.7.** (A) The two-way ANOVA (stress and treatment) results of synapsin I expression in frontal cortex: stress [*F*(1,20)=0.11, *P*>0.05], treatment [*F*(1,20)=2.11, *P*>0.05], interaction [*F*(1,20)=3.81, *P*>0.05]. Another two-way ANOVA (treatment and pretreatment) results of synapsin I expression in frontal cortex: treatment [*F*(1,20)=0.27, *P*>0.05], pretreatment [*F*(1,20)=1.86, *P*>0.05], interaction [*F(*1,20)=1.01, *P*>0.05]. (B) The two-way ANOVA (stress and treatment) results of synapsin I expression in hippocampus: stress [*F(*1,20)=23.23, *P*<0.01], treatment [*F(*1,20)=14.80, *P*<0.01], interaction [*F(*1,20)=25.66, *P*<0.01]. Another two-way ANOVA (treatment and pretreatment) results of synapsin I expression in hippocampus: treatment [*F(*1,20)=34.58, *P*<0.01], pretreatment [*F(*1,20)=69.53, *P*<0.01], interaction [*F(*1,20)=39.94, *P*<0.01]. (C) The two-way ANOVA (stress and treatment) results of synapsin I expression in amygdala: stress [*F(*1,20)=0.47, *P*>0.05], treatment [*F(*1,20)=2.29, *P*>0.05], interaction [*F(*1,20)=5.05, *P*<0.05]. Another two-way ANOVA (treatment and pretreatment) results of synapsin I expression in amygdala: treatment [*F(*1,20)=0.01, *P*>0.05], pretreatment [*F(*1,20)=3.15, *P*>0.05], interaction [*F(*1,20)=0.62, *P*>0.05]. (D) The two-way ANOVA (stress and treatment) results of synapsin I expression in hypothalamus: stress [*F(*1,20)=2.54, *P*>0.05], treatment [*F(*1,20)=0.94, *P*>0.05], interaction [*F(*1,20)=0.01, *P*>0.05]. Another two-way ANOVA (treatment and pretreatment) results of synapsin I expression in hypothalamus: treatment [*F(*1,20)=3.55, *P*>0.05], pretreatment [*F(*1,20)=4.32, *P*>0.05], interaction [*F(*1,20)=1.37, *P*>0.05].

**Supplementary Figure 1. p-mTOR (Frontal cortex)**

**
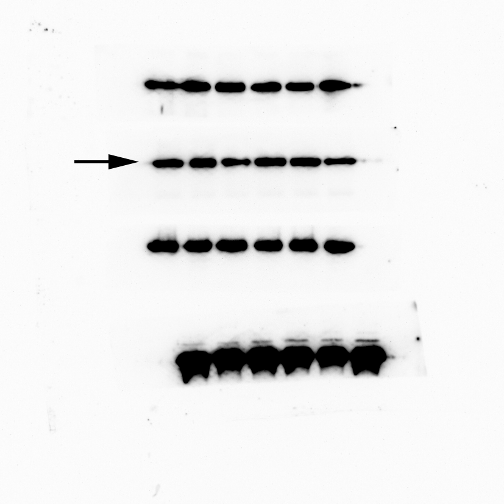
**

**Supplementary Figure 2. mTOR (Frontal cortex)**

**
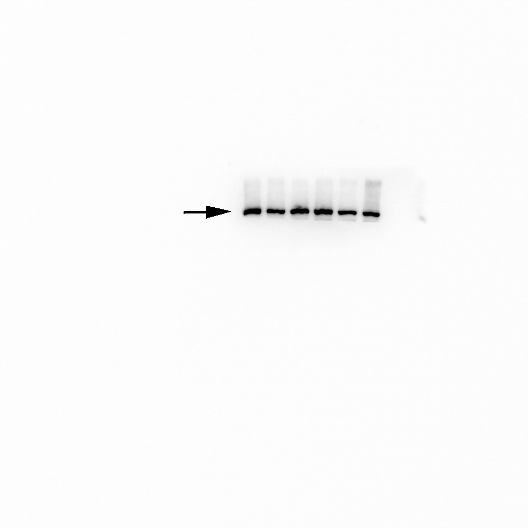
**

**Supplementary Figure 3. GAPDH (Frontal cortex)**

**
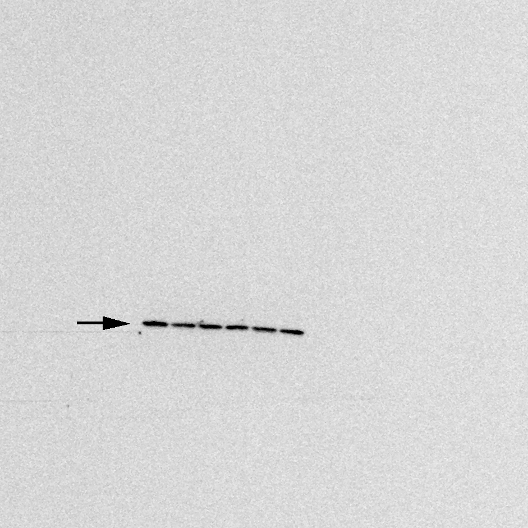
**

**Supplementary Figure 4. p-mTOR (hippocampus)**

**
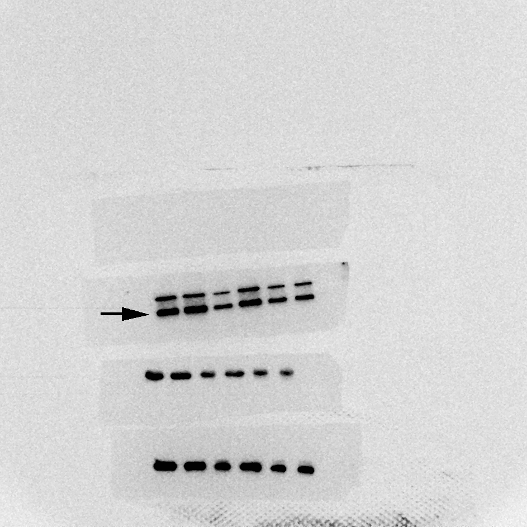
**

**Supplementary Figure 5. mTOR (Hippocampus)**

**
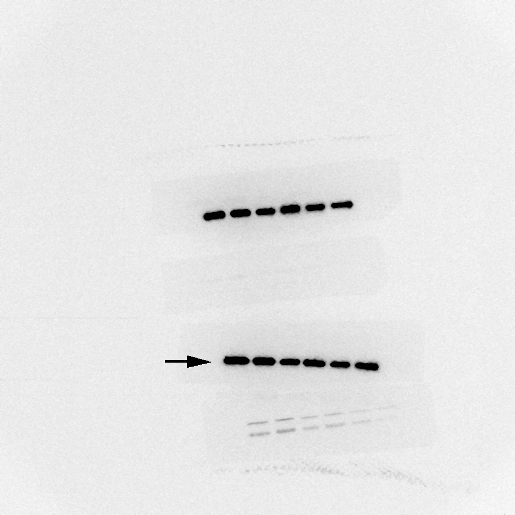
**

**Supplementary Figure 6. GAPDH (Hippocampus)**

**
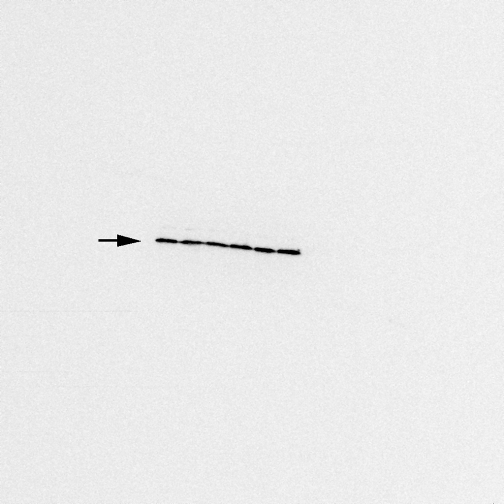
**

**Supplementary Figure 7. p-mTOR (Amygdala)**

**
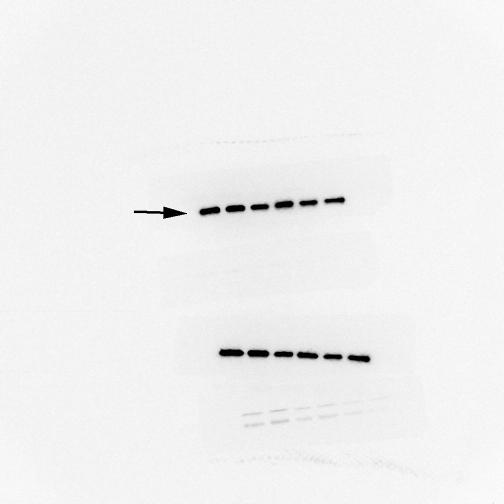
**

**Supplementary Figure 8. mTOR (Amygdala)**

**
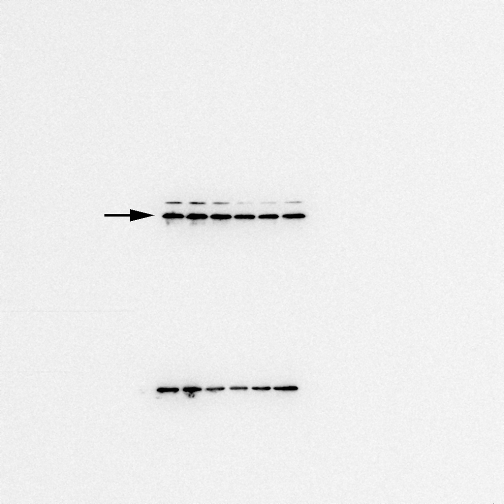
**

**Supplementary Figure 9. GAPDH (Amygdala)**

**
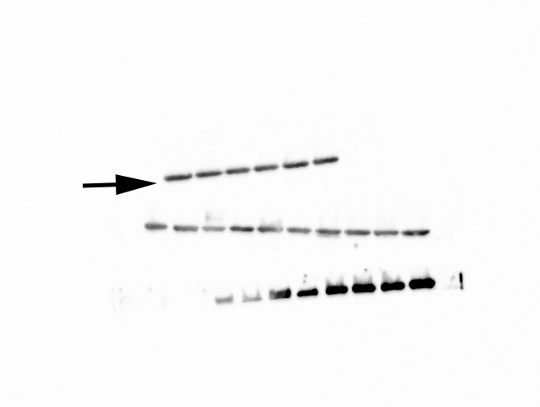
**

**Supplementary Figure 10. p-mTOR (Hypothalamus)**

**
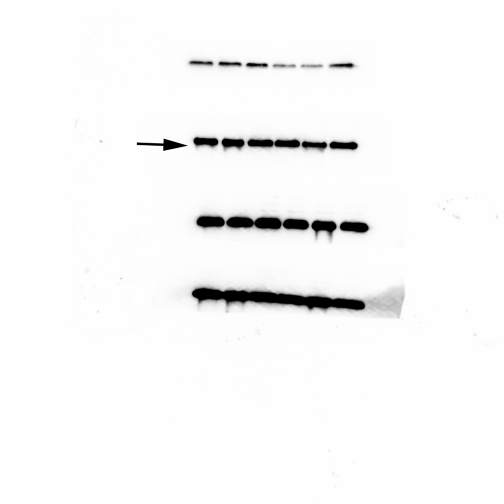
**

**Supplementary Figure 11. mTOR (Hypothalamus)**

**
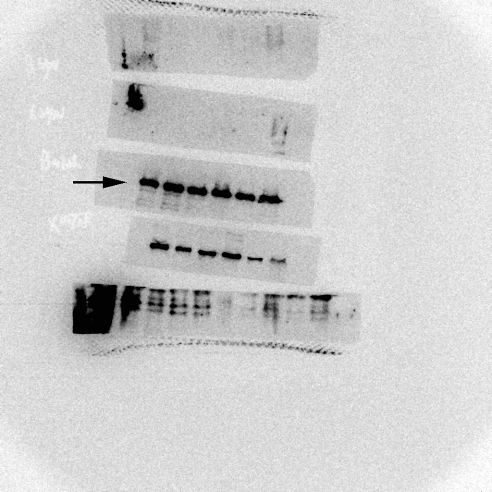
**

**Supplementary Figure 12. GAPDH (Hypothalamus)**

**
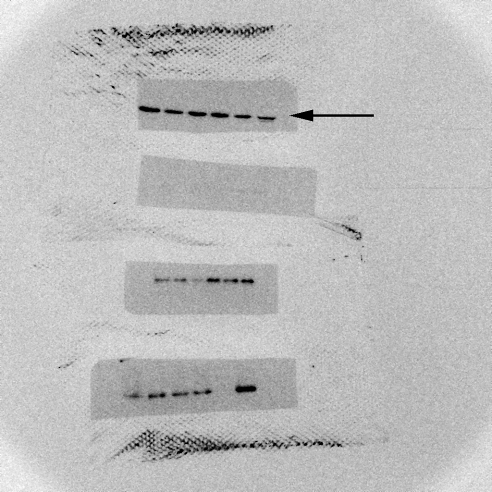
**

**Supplementary Figure 13. p-p70S6K (Frontal cortex)**

**
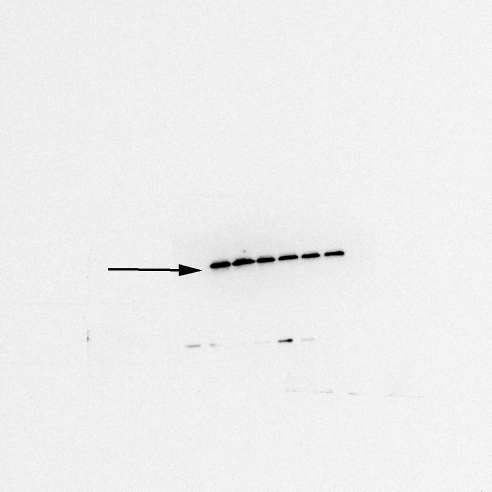
**

**Supplementary Figure 14. p70S6K (Frontal cortex)**

**
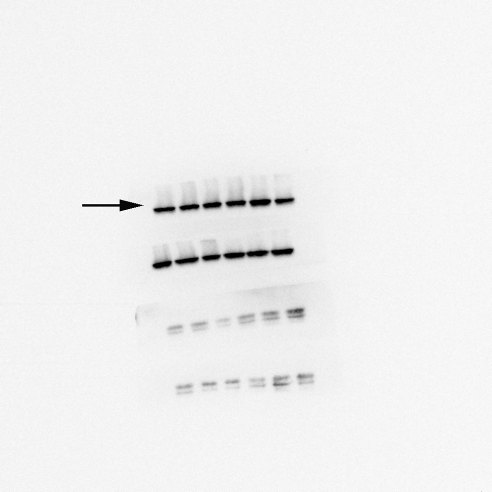
**

**Supplementary Figure 15. GAPDH (Frontal cortex)**

**
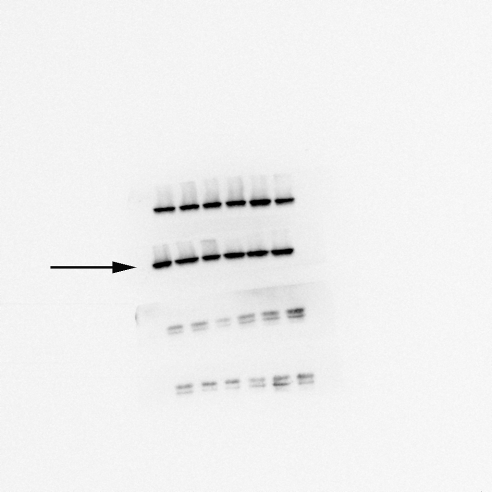
**

**Supplementary Figure 16. p-p70S6K (hippocampus)**

**
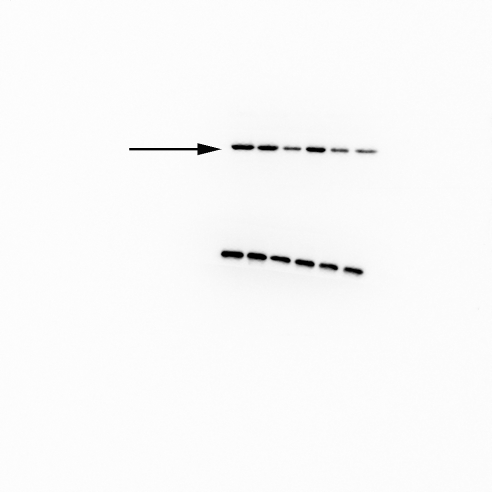
**

**Supplementary Figure 17. p70S6K (hippocampus)**

**
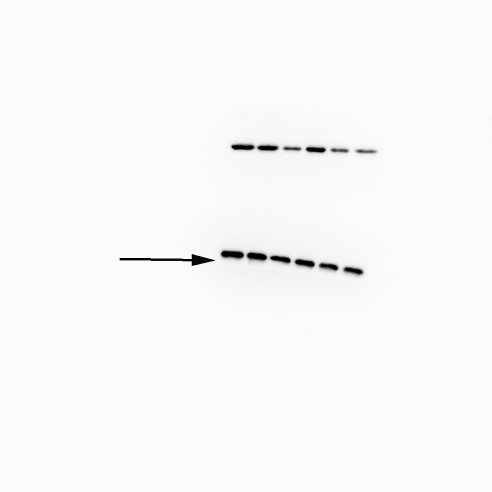
**

**Supplementary Figure 18. GAPDH (hippocampus)**

**
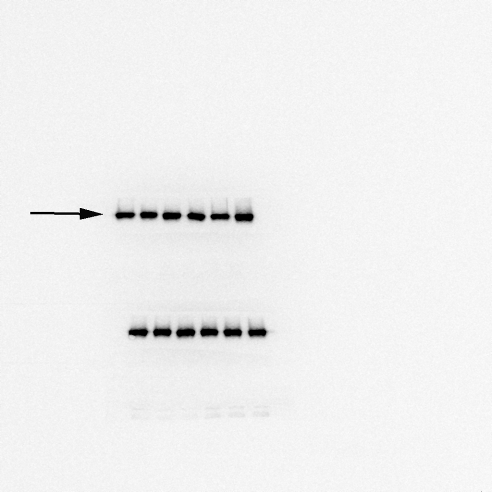
**

**Supplementary Figure 19. p-p70S6K (Amygdala)**

**
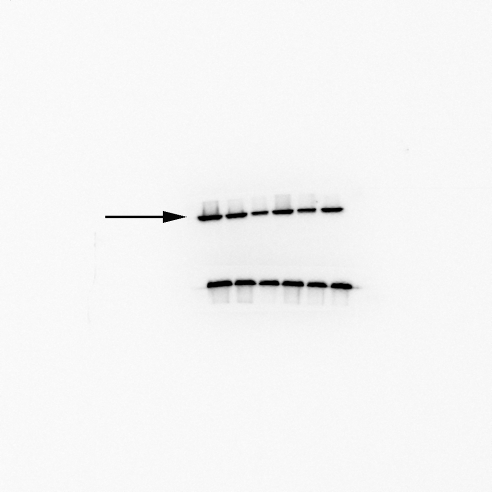
**

**Supplementary Figure 20. p70S6K (Amygdala)**

**
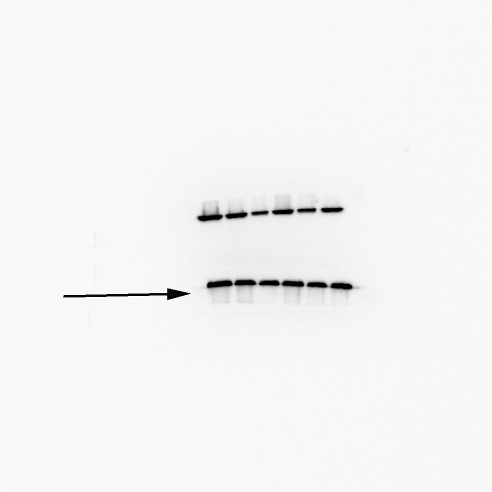
**

**Supplementary Figure 21. GAPDH (Amygdala)**

**
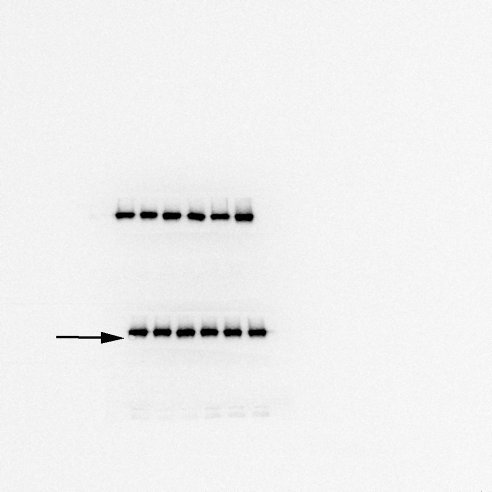
**

**Supplementary Figure 22. p-p70S6K (Hypothalamus)**

**
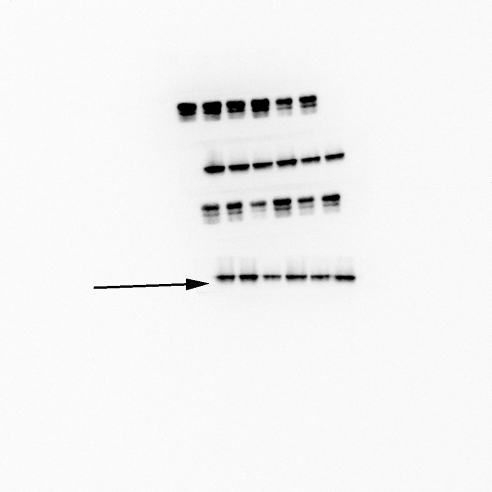
**

**Supplementary Figure 23. p70S6K (Hypothalamus)**

**
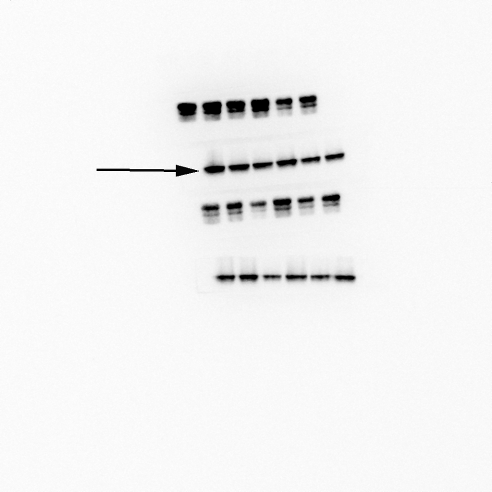
**

**Supplementary Figure 24. GAPDH (Hypothalamus)**

**
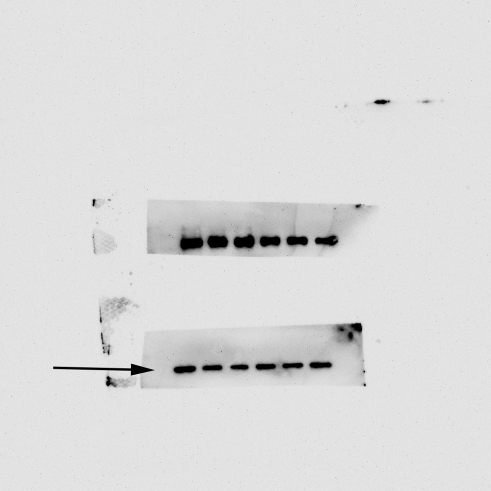
**

**Supplementary Figure 25. p-4E-BP-1 (Frontal cortex)**

**
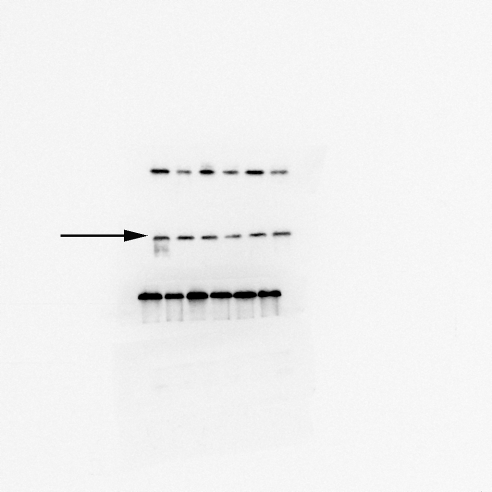
**

**Supplementary Figure 26. 4E-BP-1 (Frontal cortex)**

**
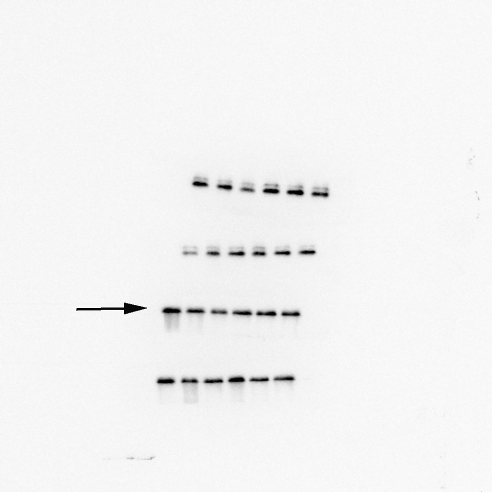
**

**Supplementary Figure 27. GAPDH (Frontal cortex)**

**
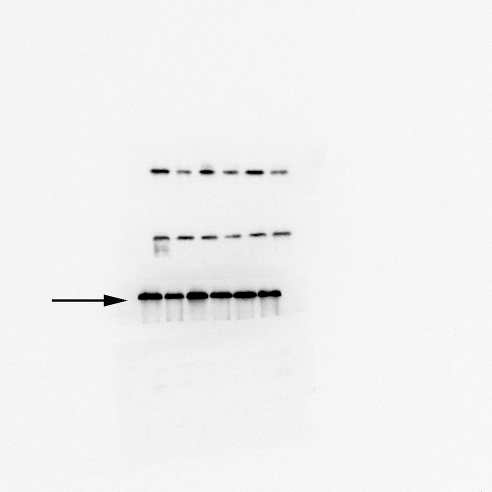
**

**Supplementary Figure 28. p-4E-BP-1 (hippocampus)**

**
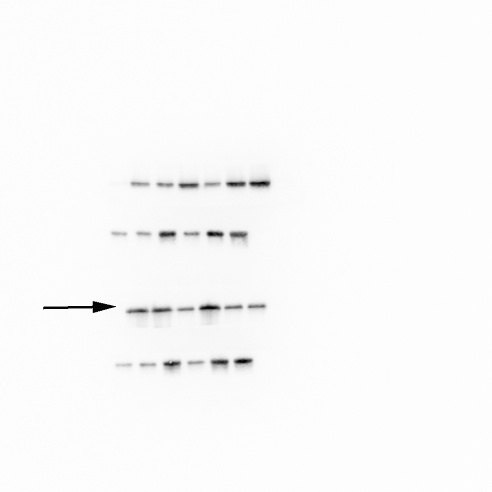
**

**Supplementary Figure 29. 4E-BP-1 (hippocampus)**

**
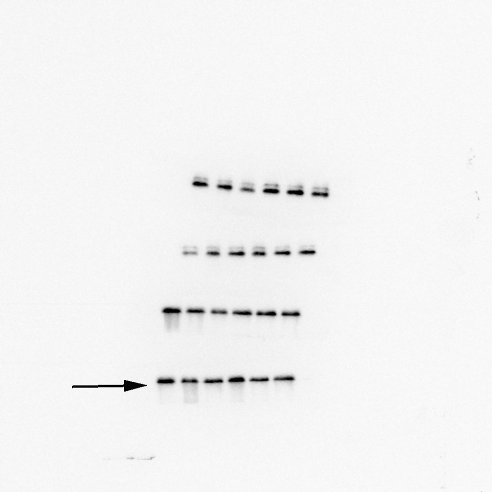
**

**Supplementary Figure 30. GAPDH (hippocampus)**

**
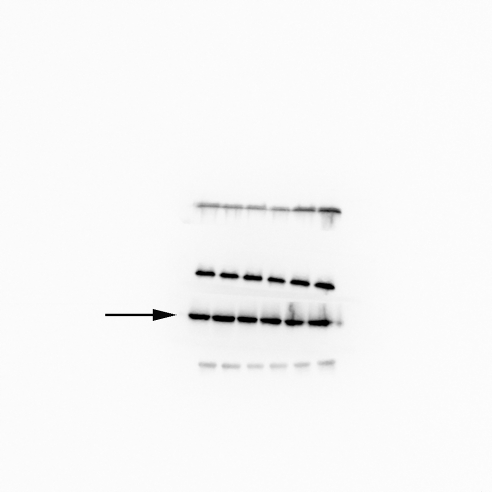
**

**Supplementary Figure 31. p-4E-BP-1 (Amygdala)**

**
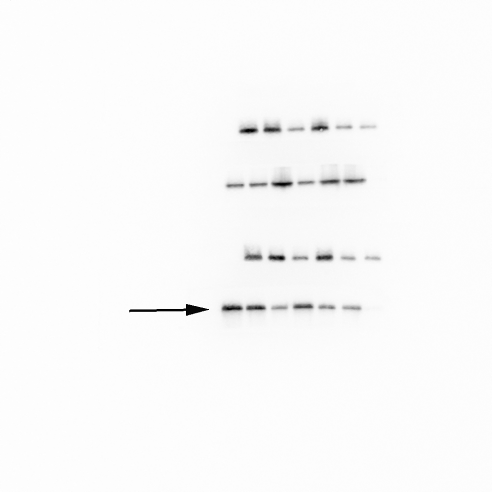
**

**Supplementary Figure 32. 4E-BP-1 (Amygdala)**

**
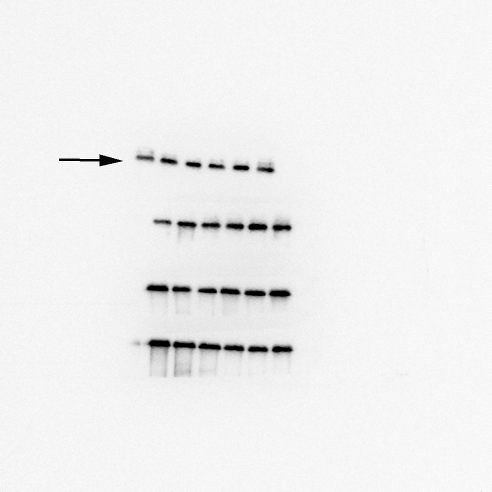
**

**Supplementary Figure 33. GAPDH (Amygdala)**

**
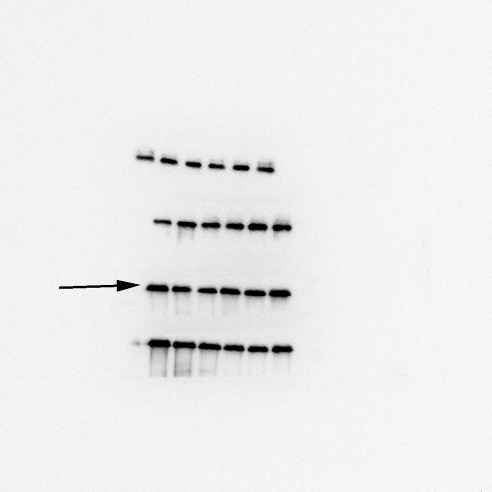
**

**Supplementary Figure 34. p-4E-BP-1 (Hypothalamus)**

**
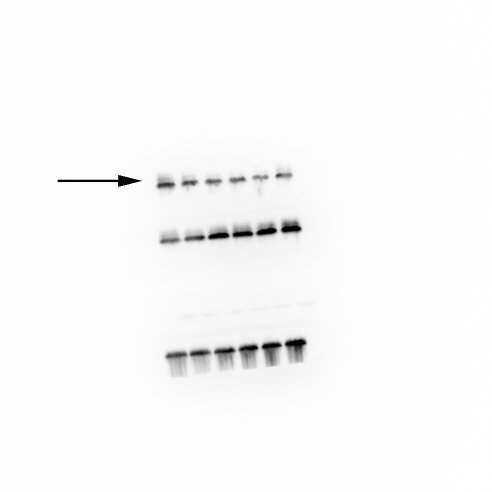
**

**Supplementary Figure 35. 4E-BP-1 (Hypothalamus)**

**
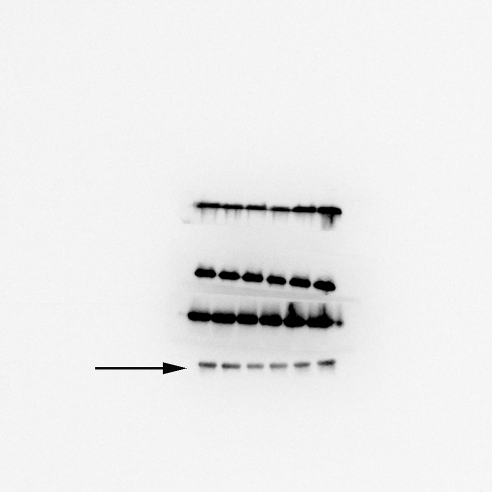
**

**Supplementary Figure 36. GAPDH (Hypothalamus)**

**
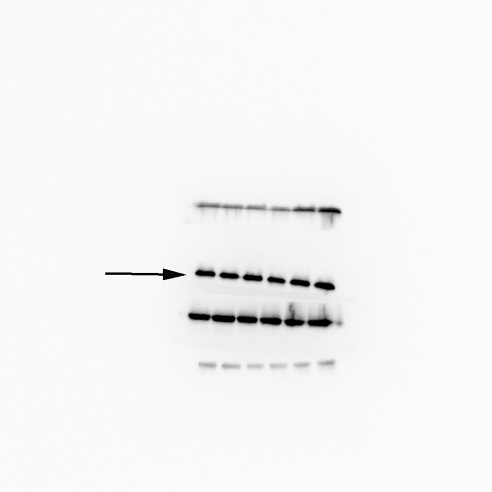
**

**Supplementary Figure 37. PSD-95 (Frontal cortex)**

**
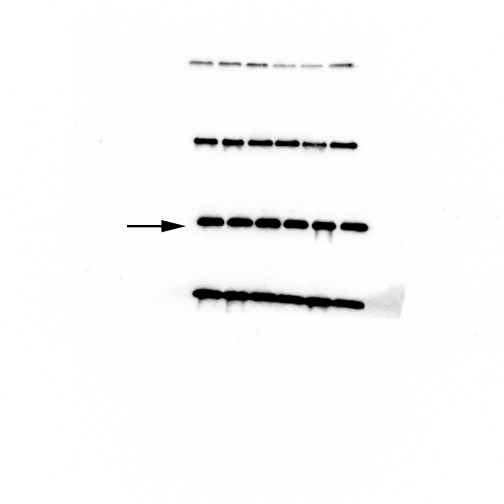
**

**Supplementary Figure 38. GAPDH (Frontal cortex)**

**
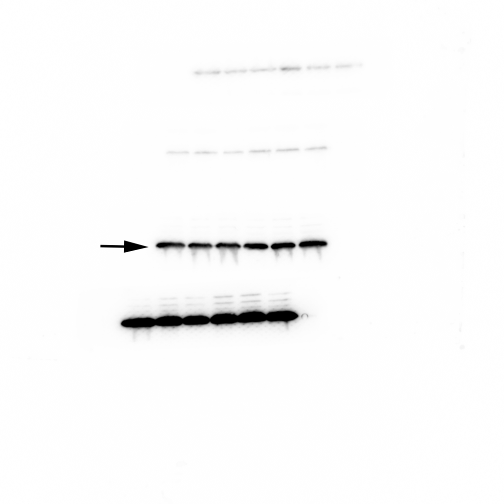
**

**Supplementary Figure 39. PSD-95 (Hippocampus)**

**
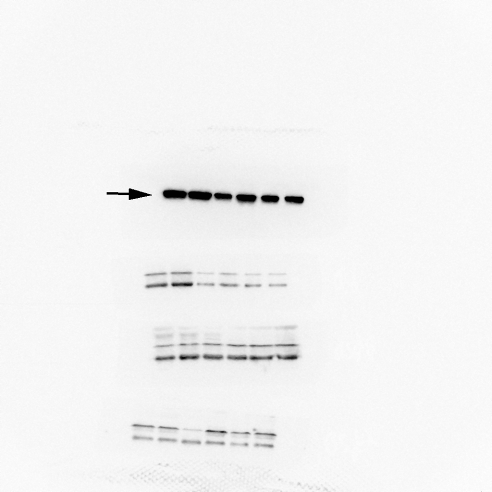
**

**Supplementary Figure 40. GAPDH (Hippocampus)**

**
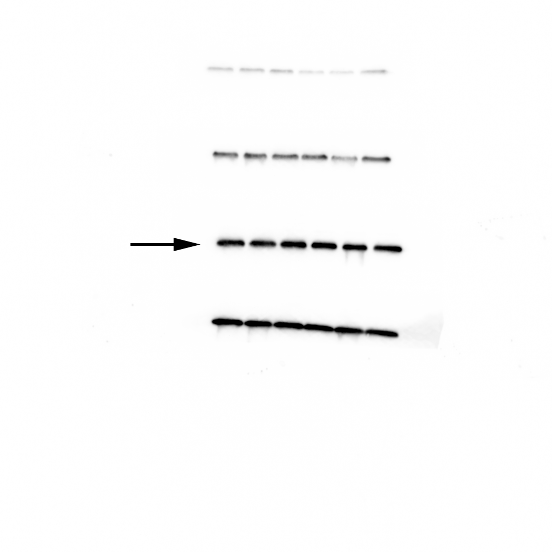
**

**Supplementary Figure 41. PSD-95 (Amygdala)**

**
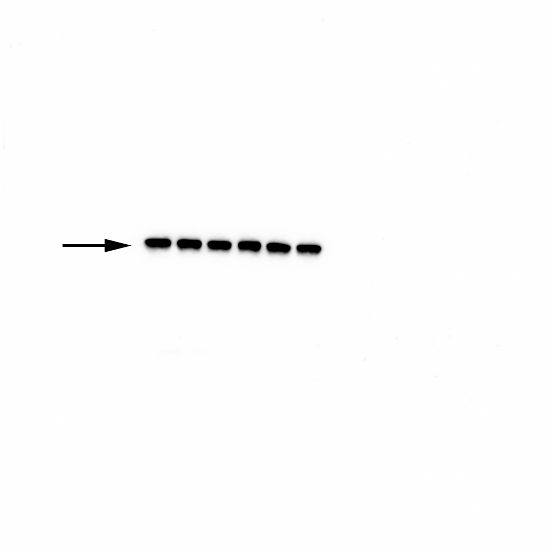
**

**Supplementary Figure 42. GAPDH (Amyglaga)**

**
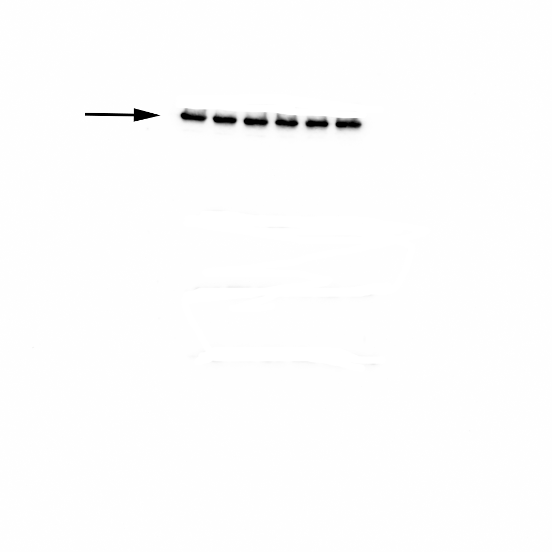
**

**Supplementary Figure 43. PSD-95 (Hypothalamus)**

**
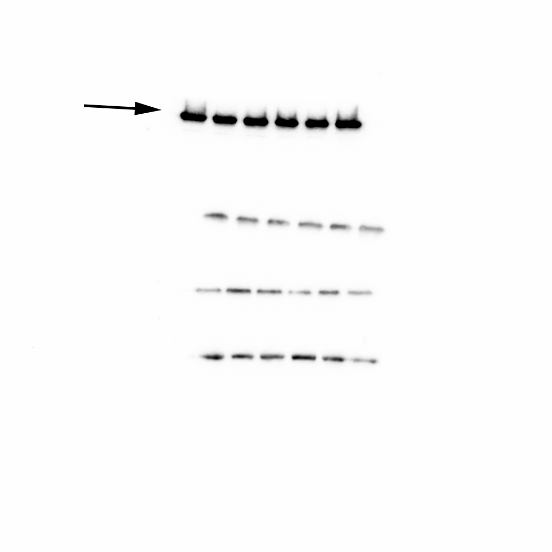
**

**Supplementary Figure 44. GAPDH (Hypothalamus)**

**
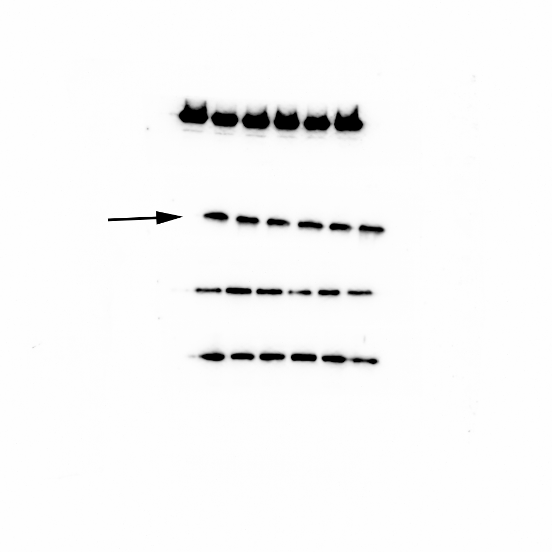
**

**Supplementary Figure 45. synapsin I (Frontal cortex)**

**
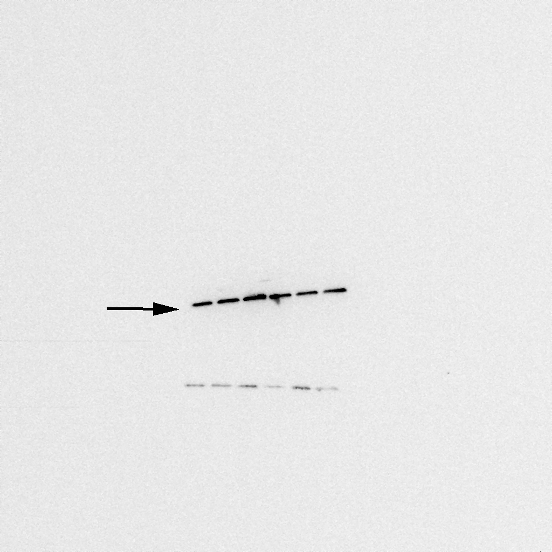
**

**Supplementary Figure 46. GAPDH (Frontal cortex)**

**
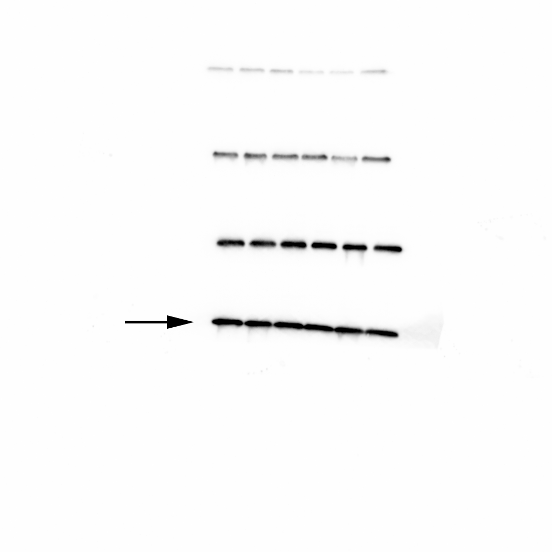
**

**Supplementary Figure 47. synapsin I (Hippocampus)**

**
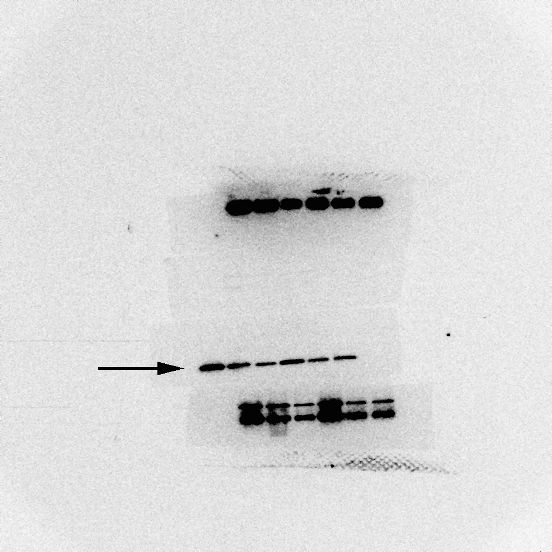
**

**Supplementary Figure 48. GAPDH (Hippocampus)**

**
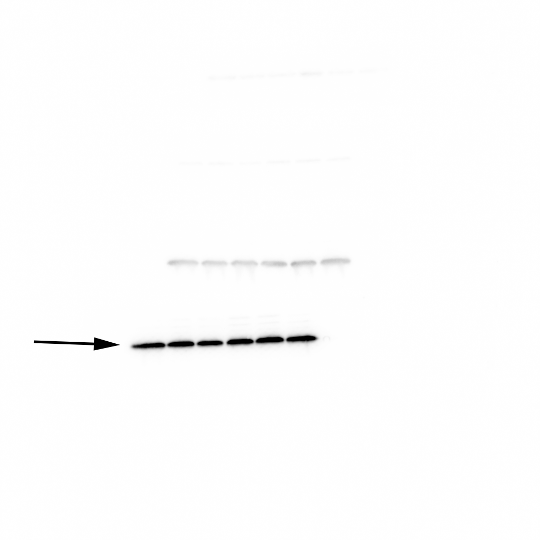
**

**Supplementary Figure 49. synapsin I (Amygdala)**

**
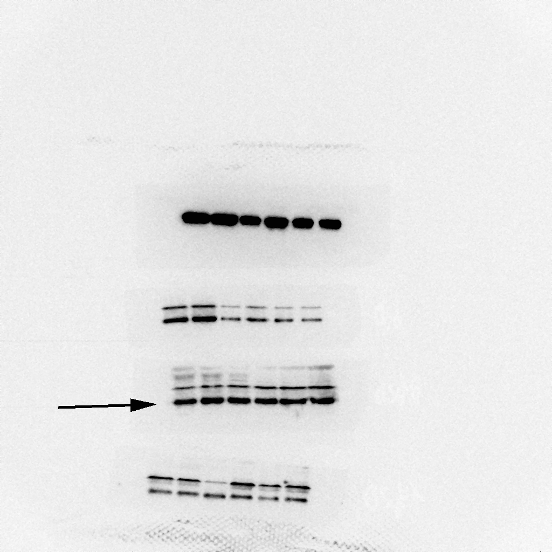
**

**Supplementary Figure 50. GAPDH (Amygdala)**

**
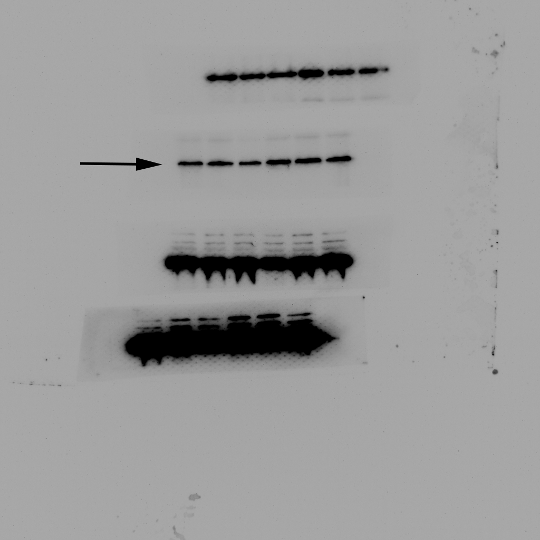
**

**Supplementary Figure 51. synapsin I (Hypothalamus)**

**
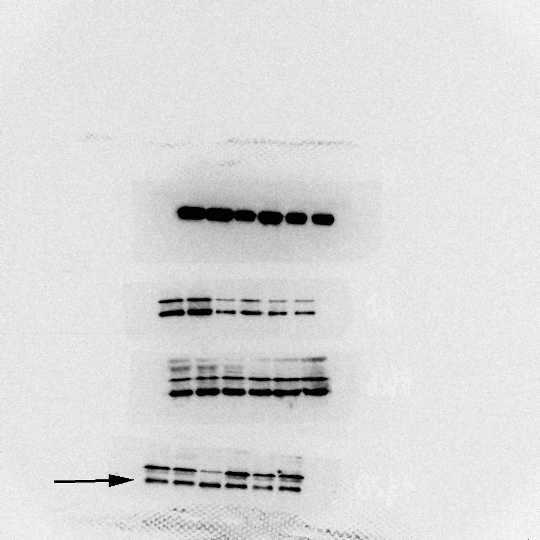
**

**Supplementary Figure 52. GAPDH (Hypothalamus)**

**
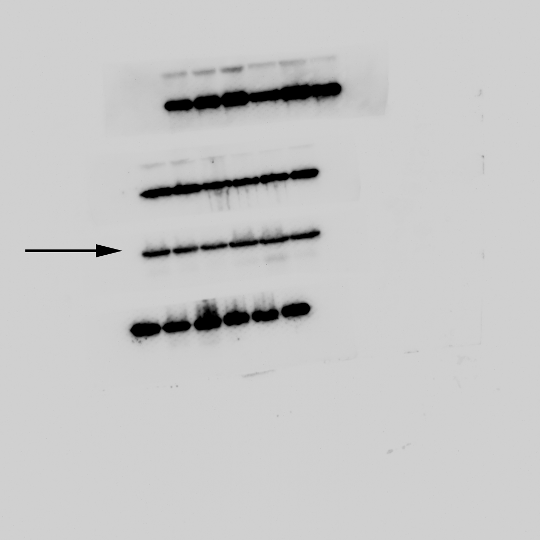
**
